# Shrinkage and Storage Stability of High‐Protein, High‐Overrun Frozen Desserts

**DOI:** 10.1111/1750-3841.70895

**Published:** 2026-02-06

**Authors:** Samantha R. VanWees, Scott A. Rankin, Richard W. Hartel

**Affiliations:** ^1^ University of Wisconsin–Madison Madison Wisconsin USA

**Keywords:** frozen desserts, high protein, meltdown, microstructure, recrystallization, shrinkage

## Abstract

Shrinkage is a significant quality issue that affects many frozen desserts and is especially prevalent in frozen desserts with increased protein and/or air content. While the mechanism of shrinkage is not well understood, this experiment sought to understand the structural and interfacial role of dairy proteins and other surface‐active ingredients in forming and stabilizing the microstructure of the product to identify physicochemical phenomena associated with air phase destabilization. The source of dairy protein and addition of mono‐ and diglycerides influenced the structure of the ice, air, fat, and serum phases, which, in combination, affected the stability of the product during melting and during temperature‐cycled storage. Frozen desserts that had a greater degree of fat destabilization collapsed more slowly at room temperature and had smaller mean ice and air cell size after 6 weeks of storage, in part due to the cooperative structuring effects of mono‐ and diglycerides, serum proteins, and micellar casein. In frozen desserts made with flexible casein proteins, severe air phase destabilization occurred and facilitated ambient collapse and shrinkage of the product during storage. Proteins and other ingredients are essential to the stepwise development of frozen dessert microstructure. Investigating the role of these structures in foam stability at room temperature and instability during storage is essential to elucidating a mechanism of shrinkage and optimizing frozen desserts to resist this defect.

**Practical Applications:**

This research highlights the roles of surface‐active ingredients in the development of microstructure during freezing and storage and demonstrates that high‐protein, high‐overrun frozen desserts can be formulated to resist recrystallization and shrinkage during storage.

## 1 Introduction

Shrinkage is a textural defect in frozen desserts characterized by the loss of volume during storage. Many researchers have associated shrinkage with the formulation, processing, and storage of frozen desserts, as these are fundamental factors in the development and stability of product structure. Fluctuating temperatures are the most common cause of shrinkage during storage (Erb 1941; Goff et al. 1995; Hankinson and Dahle 1944; Lando and Dahle 1949; Ramsey et al. 1946; Stanley et al. 1996; Trautman and Nickerson 1955); though high protein content (Frazeur and Dahle 1953; Gallegos 2021; Hankinson and Dahle 1944; Lando and Dahle 1949; Tobias 1976; Trautman and Nickerson 1955), emulsifier addition (Frazeur and Dahle 1953; Hankinson and Dahle 1944), and high air phase volume (Dubey and White 1997; Erb 1941; Hankinson and Dahle 1944; Nickerson and Tarassuk 1955; Tarassuk and Hutton 1950; Tracy 1947; Tracy and Ruehe 1929; Turan et al. 1999) have also been linked to shrinkage.

In the case of high‐overrun frozen desserts, the air phase is especially susceptible to foam destabilization and collapse. The coarsening of the air phase due to coalescence, disproportionation, or drainage is accelerated due to loss of solid‐like character in the matrix at high temperatures, and severe coarsening can result in air channel formation and shrinkage (Chang and Hartel 2002a; Turan et al. 1999; Turan and Bee 1999; VanWees et al. 2022). In addition to shrinkage, temperature fluctuations accelerate recrystallization and growth of ice crystals, resulting in an icy texture (Goff et al. 2025).

The ingredients and processing of frozen desserts affect the initial product microstructure, as well as the melting properties and storage stability. Emulsifiers are commonly added to promote partial coalescence of fat during freezing, and polysaccharide stabilizers are added to maintain the mix emulsion and prevent recrystallization during storage (Miller‐Livney and Hartel 1997). Proteins naturally present in milk or cream, or added to enhance the nutritional profile of the product, may provide some additional functionality in the mix emulsion and frozen dessert. The functionality of dairy proteins differs based on the structure of individual proteins and the interactions with other mix components such as fats, polysaccharide stabilizers, or emulsifiers. VanWees et al. (forthcoming) described the physical properties of high‐protein frozen desserts made with rehydrated milk protein concentrate (MPC), sodium caseinate (NaCN), and whey protein concentrate powders and found that the development of ice, air, and fat phases was influenced by the source of protein and the addition of mono‐ and diglycerides.

The complexity of ice, air, fat, and serum phases in frozen desserts renders these products kinetically and thermodynamically unstable. The small ice crystal and air cell size distributions produced during continuous freezing have been shown to prevent (Chang and Hartel 2002a; Sofjan and Hartel 2004; Turan and Bee 1999) and promote (Erb 1941; Iverson 1947; Tracy 1947) shrinkage, while destabilized fat networks generally maintain the stability of the foam during melting and storage fat destabilization and foam stabilization (Goff et al. 1999b; Jiang et al. 2019; Pelan et al. 1997; Turan et al. 1999). Understanding the size distributions of discrete structural phases in the product, as well as the co‐development of structures and their roles in foam stability, is critical to identifying causes and mechanisms of shrinkage in multiphase frozen desserts. While it is easily identified as a loss of product volume, no common cause or mechanism of shrinkage has been identified. The objective of this work was to understand the influence of proteins, mono‐ and diglycerides (MDGs), and overrun on the formation and destabilization of structure in frozen desserts and to identify the role of structural components in foam collapse at room temperature (i.e., during melting) and frozen storage (i.e., shrinkage).

## 2 Materials and Methods

Powdered MPC, NaCN, or whey protein isolate (WPI) was used to formulate a high‐protein frozen dessert mix with and without added MDGs. Frozen desserts were manufactured with 100% or 150% overrun, then stored for up to 6 weeks to investigate the effects of formulation, overrun, and storage time on microstructural and physical properties.

### 2.1 Materials

Powdered MPC was obtained from Idaho Milk Products (Jerome, ID), and NaCN was obtained from Milk Specialties Global (Eden Prairie, MN). WPI and the milk mineral blend were obtained from Glanbia Nutritionals (Twin Falls, ID). Anhydrous milk fat (AMF) and crystalline lactose were obtained from Grassland Dairy (Greenwood, WI); sucrose was obtained from Domino Foods (Yonkers, NY); and the MDGs and stabilizer blend of guar gum, locust bean gum, and carrageenan were obtained from Danisco (New Century, KS).

## 3 Methods

### 3.1 Frozen Dessert Mix Production

All mixes were formulated to contain 12.0% fat, 13.3% milk solids nonfat (6.0% protein, 6.3% lactose, and 1.0% ash), 14.5% sucrose, 0.2% stabilizer, and 0.0% or 0.15% MDGs. The target calculated freezing point (−2.60°C) was achieved by varying the concentration of dairy ingredients and water. Details regarding the composition of the materials and mixes can be found in VanWees et al. (forthcoming).

Two batches (22–25 kg each) were made in duplicate for each formulation. Melted AMF, sucrose, lactose, milk minerals, stabilizer, and MDGs (if present) were combined with 50% of the mix water and heated to 85°C using a jacketed mixer (Stephan Food Processing Machinery, Hamelin, Germany) to dissolve sugars and hydrate the hydrocolloid stabilizers. The mixture was cooled to 70°C using chilled water, then combined with a dispersion of protein powder and the remaining mix water that had been previously rehydrated (42°C for 60 min). The mix was homogenized using a two‐stage homogenizer (Manton‐Gaulin MFG Co. Inc., Everett, MA) set to 13.8 MPa on the first stage and 3.4 MPa on the second stage, then cooled to 20°C before being aged overnight at 4°C.

### 3.2 Freezing and Hardening

All frozen desserts were frozen using a continuous freezer (Hoyer Frigus KF 80 F; Tetra Pak Hoyer Inc., Aarhus, Denmark). Air input was varied to achieve an overrun of 100.0% (±0.43%) or 150.1% (±0.49%), while dasher speed (500 RPM), cylinder pressure (358.2 kPa), and draw temperature (−4.68°C ± 0.33°C) were maintained across treatments. To validate product overrun, containers (103.5 mL) were filled with mix or frozen desserts, and overrun was calculated as the difference between the mass of the mix and the mass of the frozen product, relative to the mass of the frozen product at an equivalent volume. At least 48 containers of frozen dessert were packaged (118.3 mL containers), then immediately hardened at −29°C for at least 2 h.

### 3.3 Storage

Following hardening, frozen desserts were stored at −29°C for 12–16 h, then transferred to a refrigerated cabinet for extended storage. The temperature of the cabinet was set to −12.6°C, and the internal temperature cycled from −10.8°C to −14.4°C every 30 min. Selected samples were removed at 2, 4, and 6 weeks of storage for analysis.

### 3.4 Frozen Dessert Analyses

#### 3.4.1 Ice Crystal Size Distribution

Ice crystals were observed using the method of Donhowe et al. (1991). An optical microscope (Accu‐scope 3000‐LED; Accu‐scope, Commack, NY) was used to photograph ice crystals at −15°C. Frozen desserts were tempered at −20°C overnight, then at −15°C for 30 min in a refrigerated glovebox immediately prior to observation. Chilled dispersant (50% pentanol, 50% kerosene) was added to aid visualization of ice crystals, and approximately 20 images were captured using a Moticam 3+ microscope digital camera (Moticam, Kowloon, Hong Kong). At least 300 ice crystals were manually traced using Microsoft Paint and enumerated using Image Pro Plus (Version 7.0, Media Cybernetics 2009, Rockville, MD).

#### 3.4.2 Air Cell Size Distribution

Air cells were measured using the method of Chang and Hartel (2002b). Prior to observation, frozen desserts were tempered at −20°C for overnight, then 60 min at −15°C in a refrigerated glovebox. A core sample of frozen dessert was placed in the center of a fabricated well slide and covered with a cover slip. The temperature of the glovebox was slowly raised to −6°C (1°C–2°C per minute) to promote visualization of air cells, and approximately 10 images were captured using a microscope digital camera. Image Pro Plus was used to trace and enumerate at least 300 air cells per sample.

#### 3.4.3 Fat Globule Size Distribution

The particle size distributions of emulsified fat globules in frozen dessert mix and fat globule clusters in melted frozen desserts were measured using laser light scattering (Malvern Mastersizer 3000; Malvern Instruments Ltd., Worcestershire, UK). Details of the measurement method can be found in VanWees et al. (forthcoming). One peak, representing emulsified fat globules, was visible in the frozen dessert mix, while melted frozen desserts displayed two peaks: one representing individual milkfat globules (0.6–1.2 µm) and one representing clusters of destabilized fat (10–100 µm). The ratio of the volume percentage of destabilized fat in the melted frozen dessert compared to the volume percentage of fat present in the mix was used to calculate the percentage of destabilized fat. Samples were viewed using optical microscopy (Nikon Labophot‐2 microscope, Tokyo, Japan) to confirm the presence of destabilized fat.

#### 3.4.4 Melting Behavior

Frozen desserts were removed from storage and tempered at −20°C overnight prior to analysis using a drip‐through test (Bolliger et al. 2000). The packaging was removed from the container, and the weight of the frozen dessert was recorded before the slab (4.0 mm in height, 7.4 mm in diameter) was placed on a wire mesh screen (three openings per centimeter), positioned above a beaker to collect drops, and allowed to melt at ambient temperature (22°C) for 360 min or until melting reached a plateau. The mass of the sample that had dripped through the mesh was recorded automatically every 60 s, and the time to reach the first drop was recorded manually as the induction time. Drip‐through rate was calculated as the slope of the relative quantity (%) of dripped‐through material as a function of time. Each frozen dessert was analyzed in triplicate.

#### 3.4.5 Shrinkage

**FIGURE 1:**
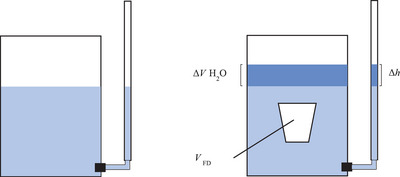
Graphical representation of the apparatus used to measure the volume of frozen desserts.

Shrinkage was calculated by evaluating the change in product volume during storage relative to the initial product volume. An overflow volume displacement method has previously been used by researchers (Dubey and White 1997; Gallegos 2021) to quantify volume loss during storage. This work utilized the principle of volume displacement and quantified the change using a straight‐walled container and an external level glass apparatus, shown schematically in Figure 1. All measurements were conducted at ambient refrigeration temperatures.

The frozen dessert was removed from its packaging using a chilled knife at −29°C, then submerged in chilled water (4°C) for 15 s using chilled tongs. The height of the water displaced was measured using an external level glass (Δ*h*) and converted to volume using a standard curve (*V*_H2O_). The volume of water displaced was equal to the volume of frozen dessert (*V*_FD_) according to Archimedes’ principle. Frozen desserts were measured at each sampling time. Shrinkage was calculated using Equation (1), where *V_t_* represents the volume at a given storage time *t* and *V*_0_ represents the initial (unshrunken) product volume. At least four containers were measured for each variable.

(1) $$\mathrm{Shrinkage}\;\left(\%\right)=\left|\frac{V_{t}-\;V_{0}}{V_{0}}\right|.$$

### 3.5 Statistics

Statistical analysis was conducted using statistical software (R Version 3.3; Posit, Boston, MA). One‐way ANOVA with Tukey's HSD post hoc analyses (*α* = 0.05) was used to evaluate main effects of protein source, MDG addition, and overrun. Multiple linear regression was used to identify the effects of storage time on structural and physical properties as a function of formulation, as well as the effects of microstructure (mean ice crystal size, mean air cell size, fat destabilization) on melting behavior and shrinkage. Associations between structural and physical properties were evaluated using principal component analysis.

### 3.6 Results and Discussion

#### 3.6.1 Ice Crystal Size and Distribution

**FIGURE 2:**
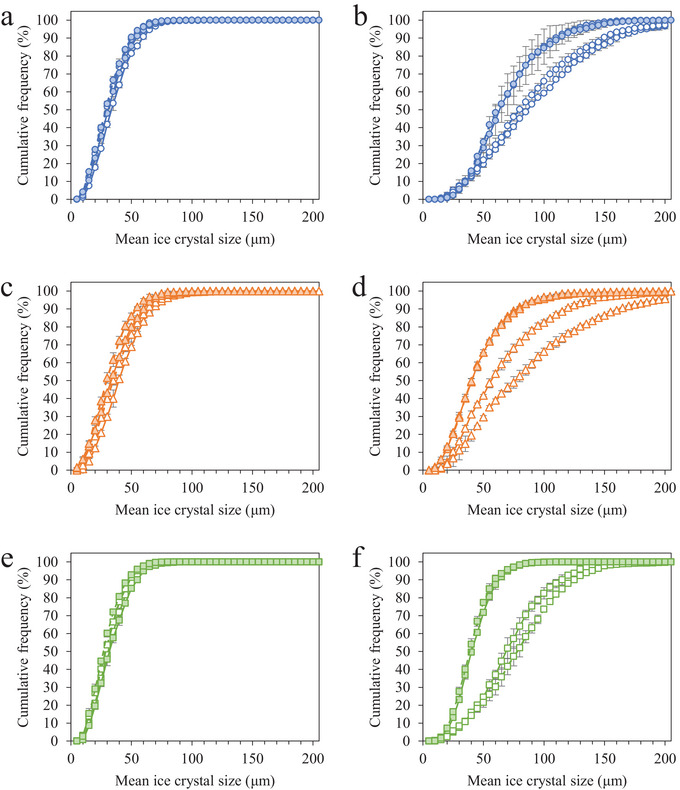
Ice crystal size distributions for frozen desserts made with 6% milk protein concentrate (a, b), sodium caseinate (c, d), or whey protein isolate (e, f) after 0 weeks (a, c, e) and 6 weeks (b, d, f) of temperature‐cycled storage. Mono‐ and diglyceride addition of 0.0% (open symbols) or 0.15% (filled symbols) and overrun of 100% (solid lines) or 150% (dashed lines) are shown. Error bars represent the standard deviation of mean values measured in duplicate.

Storage at elevated, fluctuating temperatures promoted recrystallization and ice crystal growth in the high‐protein, high‐overrun frozen desserts, and the mean ice crystal size at a given time was affected by protein source, MDG addition, and overrun. The mean ice crystal size prior to storage ranged from 28.7 to 41.9 µm, and after 6 weeks of storage, it ranged from 39.8 to 94.3 µm (Table 1). In general, the mean ice crystal size increased linearly during storage, and the mean ice crystal size distributions became broader as storage time increased for all frozen desserts, indicative of ice recrystallization (Figure 2).

**TABLE 1:** Mean ice crystal size of frozen desserts made with 6% milk protein concentrate (MPC), sodium caseinate (NaCN), or whey protein isolate (WPI) with varying levels of mono‐ and diglycerides (MDGs) and overrun (OR) measured during temperature‐cycled storage.

Mean ice crystal size (µm)						
Protein source	MDGs (%)	OR (%)	Storage time (weeks)			
			0	2	4	6
MPC	0.0	100	35.7 ± 0.54^a,A,x^	61.8 ± 0.14^a,A,x^	80.0 ± 0.85^a,A,x^	94.3 ± 1.82^a,A,x^
		150	31.3 ± 0.49^a,A,y^	63.6 ± 3.22^a,A,x^	83.4 ± 0.28^a,A,x^	88.1 ± 3.21^a,A,x^
	0.15	100	32.8 ± 0.03^a,B,x^	45.2 ± 1.33^a,B,x^	58.1 ± 0.99^a,B,x^	68.8 ± 7.31^a,B,x^
		150	30.2 ± 0.09^a,A,y^	47.1 ± 0.80^a,B,x^	55.3 ± 0.19^a,B,x^	69.0 ± 4.05^a,B,x^
NaCN	0.0	100	41.9 ± 0.49^b,A,x^	55.9 ± 0.63^b,A,x^	59.3 ± 3.05^b,A,x^	65.8 ± 0.29^b,A,x^
		150	37.6 ± 0.72^b,A,y^	56.9 ± 1.67^b,A,x^	64.9 ± 4.32^b,A,y^	87.2 ± 3.13^a,A,y^
	0.15	100	40.3 ± 0.23^b,B,x^	48.8 ± 0.83^b,B,x^	48.9 ± 0.65^b,B,x^	50.3 ± 0.67^b,B,x^
		150	36.5 ± 0.93^b,A,y^	45.0 ± 0.04^a,B,y^	45.1 ± 0.22^b,B,y^	51.2 ± 0.89^b,B,x^
WPI	0.0	100	32.5 ± 0.73^c,A,x^	61.9 ± 0.93^a,A,x^	56.5 ± 1.41^b,A,x^	71.3 ± 2.23^b,A,x^
		150	30.1 ± 0.46^a,A,y^	59.5 ± 1.65^b,A,x^	56.5 ± 0.98^c,A,x^	79.3 ± 1.26^b,A,y^
	0.15	100	33.7 ± 0.81^a,A,x^	42.8 ± 1.3^a,B,x^	38.2 ± 0.59^c,B,x^	42.0 ± 0.25^c,B,x^
		150	28.7 ± 0.20^c,B,y^	40.9 ± 0.03^b,B,x^	33.7 ± 0.46^c,B,y^	39.8 ± 0.80^c,B,x^

*Note*: Mean ± SD are shown. Superscript letters a, b, and c denote significant differences with protein source; superscript letters A and B denote significant differences by MDG addition; and superscript letters x and y denote significant differences by overrun. Values in the same column sharing a letter are not significantly different at *α* = 0.05.

The mean size of ice crystals in frozen desserts made with NaCN was significantly larger prior to storage compared to those made with MPC or WPI. This was likely due to a slight increase in draw temperature (+0.63°C) for frozen desserts made with NaCN, which has been shown to affect the ice phase volume and mean ice crystal size after hardening (Amador et al. 2017; Inoue et al. 2012). The addition of 0.15% MDGs had minimal effect on mean ice crystal size for each protein source and overrun prior to storage, whereas mean ice crystal size decreased for all frozen desserts as overrun increased.

For all protein sources, the mean ice crystal size increased linearly as a function of storage time, and the addition of 0.15% MDGs significantly reduced the size of ice crystals at a given time as well as the rate of growth during storage for frozen desserts at both 100% and 150% overrun. Overrun had no effect on the rate of ice crystal growth for frozen desserts made with MPC and 0.0% MDGs or 0.15% MDGs. Increasing the overrun to 150% resulted in a higher mean ice crystal size and a faster rate of ice crystal growth in frozen desserts made with NaCN.

At each week of storage, the frozen desserts made with WPI, 0.15% MDGs, and 150% overrun had the lowest mean ice crystal size of all formulations and were the only frozen desserts not to exceed the 50 µm threshold for sensory coarseness after 6 weeks of temperature‐cycled storage. Frozen desserts made with WPI and 0.0% MDGs had low fat destabilization and low aqueous protein concentration; thus, the extent and rate of recrystallization were higher than when 0.15% MDGs were added.

#### 3.6.2 Air Cell Size and Distribution

**FIGURE 3:**
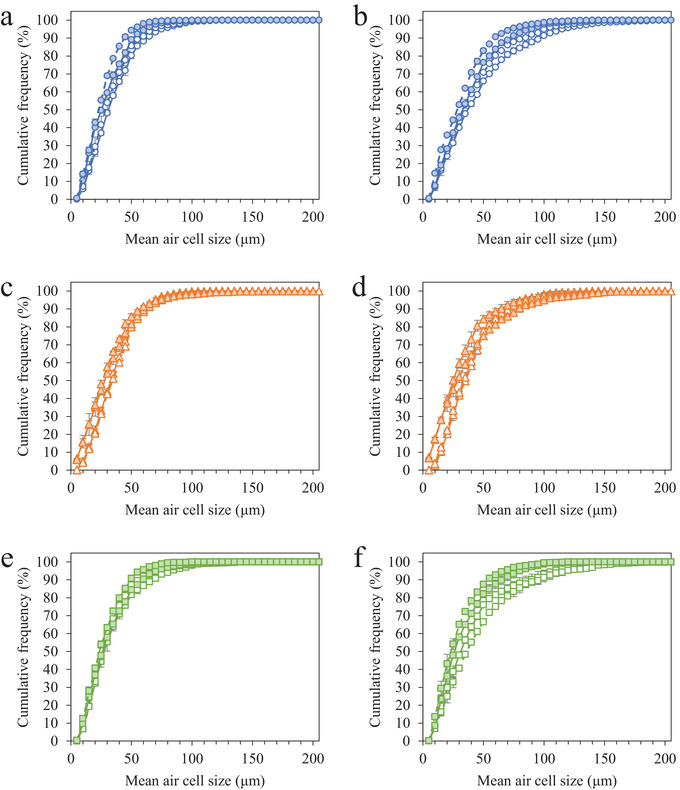
Air cell size distributions for frozen desserts made with 6% milk protein concentrate (a, b), sodium caseinate (c, d), or whey protein isolate (e, f) after 0 weeks (a, c, e) and 6 weeks (b, d, f) of temperature‐cycled storage. Mono‐ and diglyceride addition of 0.0% (open symbols) or 0.15% (filled symbols) and overrun of 100% (solid lines) or 150% (dashed lines) are shown. Error bars represent the standard deviation of mean values measured in duplicate.

Destabilization of the air phase is a known precursor to shrinkage in frozen desserts. External temperature fluctuations introduce interfacial stresses and weaken the integrity of the surrounding phases, so disproportionation, coalescence, and drainage of the foam are favorable (Goff et al. 1999b). Like ice crystals, the mean air cell size increased due to coalescence, disproportionation, and drainage, and the size distribution broadened with time, but to a much lesser extent than was observed in the ice crystals (Table 2). Air cells and ice crystals had similar mean sizes prior to storage, but the extent of air cell coarsening was significantly less than that of ice crystals, demonstrating the complexity of stabilizing the frozen foams during temperature‐cycled storage (Figure 3). Comparing the three protein sources after 6 weeks of cycled storage, frozen desserts made with NaCN had the smallest mean size when no MDGs were added, whereas frozen desserts made with WPI had the smallest mean size when 0.15% MDGs were added. In general, increasing the overrun from 100% to 150% slowed the growth of air cells during storage, but the results were varied for each protein source and MDG addition.

**TABLE 2:** Mean air cell size of frozen desserts made with 6% milk protein concentrate (MPC), sodium caseinate (NaCN), or whey protein isolate (WPI) with varying levels of mono‐ and diglycerides (MDGs) and overrun (OR) measured during temperature‐cycled storage.

Mean air cell size (µm)						
Protein source	MDGs (%)	OR (%)	Storage time (weeks)			
			0	2	4	6
MPC	0.0	100	35.0 ± 0.55^a,A,x^	39.5 ± 0.04^a,A,x^	39.4 ± 0.59^ab,A,x^	46.3 ± 0.46^a,A,x^
		150	32.3 ± 0.45^a,A,y^	36.9 ± 0.09^a,A,y^	40.0 ± 0.63^a,A,x^	41.1 ± 0.12^a,A,y^
	0.15	100	28.9 ± 0.53^a,B,x^	33.0 ± 0.62^a,B,x^	38.2 ± 2.19^a,A,x^	37.6 ± 0.04^a,B,x^
		150	25.1 ± 0.03^a,B,y^	33.0 ± 0.63^a,B,x^	35.0 ± 0.75^a,B,y^	32.0 ± 0.46^a,B,y^
NaCN	0.0	100	35.4 ± 0.74^a,A,x^	37.2 ± 0.20^b,A,x^	37.0 ± 0.22^a,A,x^	38.6 ± 0.42^b,A,x^
		150	36.7 ± 0.28^b,A,x^	38.5 ± 0.32^b,A,y^	39.7 ± 0.13^a,A,y^	38.4 ± 0.19^b,A,x^
	0.15	100	36.8 ± 2.12^b,A,x^	37.1 ± 0.95^b,A,x^	41.2 ± 0.66^b,B,x^	36.2 ± 2.35^a,B,x^
		150	35.3 ± 0.58^b,A,x^	36.0 ± 0.46^b,B,y^	38.5 ± 1.15^b,A,y^	41.5 ± 0.49^b,B,y^
WPI	0.0	100	34.0 ± 1.35^a,A,x^	36.9 ± 0.06^b,A,x^	40.2 ± 0.12^b,A,x^	40.4 ± 0.67^b,A,x^
		150	31.2 ± 0.19^a,A,y^	38.6 ± 0.06^b,A,y^	38.1 ± 0.46^a,A,y^	46.0 ± 0.86^c,A,y^
	0.15	100	31.1 ± 0.16^a,B,x^	33.6 ± 0.01^a,B,x^	34.5 ± 0.41^c,B,x^	32.6 ± 1.21^b,B,x^
		150	27.2 ± 0.33^a,B,y^	34.8 ± 0.58^c,B,y^	31.9 ± 1.80^c,B,y^	28.3 ± 0.72^c,B,y^

*Note*: Mean ± SD are shown. Superscript letters a, b, and c denote significant differences with protein source; superscript letters A and B denote significant differences by MDG addition; and superscript letters x and y denote significant differences by overrun. Values in the same column sharing a letter are not significantly different at *α* = 0.05.

Prior to storage, frozen desserts made with NaCN had the highest mean air cell size, possibly due to the aforementioned increase in draw temperature (Caillet et al. 2003; Eisner et al. 2005; Hernández Parra et al. 2018; Inoue et al. 2008; Rohenkohl and Kohlus 1999). Mean air cell size decreased with MDG addition and overrun for frozen desserts made with MPC and WPI, but there were no effects on those made with NaCN.

Storage time, MDG addition, and overrun significantly affected the mean size of air cells in frozen desserts made with MPC. The addition of 0.15% MDGs significantly reduced the mean size of air cells at a given storage time, but the rate of increase with time was unaffected by MDG addition or overrun. For frozen desserts made with NaCN, there were no significant effects of MDG addition or overrun on the mean air cell size or distribution at a given time, nor was the overall rate of air cell growth affected. Frozen desserts made with NaCN had the highest mean air cell size prior to storage and the lowest increase in air cell size across the three protein sources. In frozen desserts made with WPI, the addition of 0.15% MDGs significantly decreased the mean size of air cells and the rate of air cell growth during storage. An increase in overrun generally decreased the mean air cell size, but this was not consistent or significant. The mean air cell size increased significantly with storage, and the rate and extent were the lowest of the three protein sources studied.

#### 3.6.3 Fat Destabilization

**FIGURE 4:**
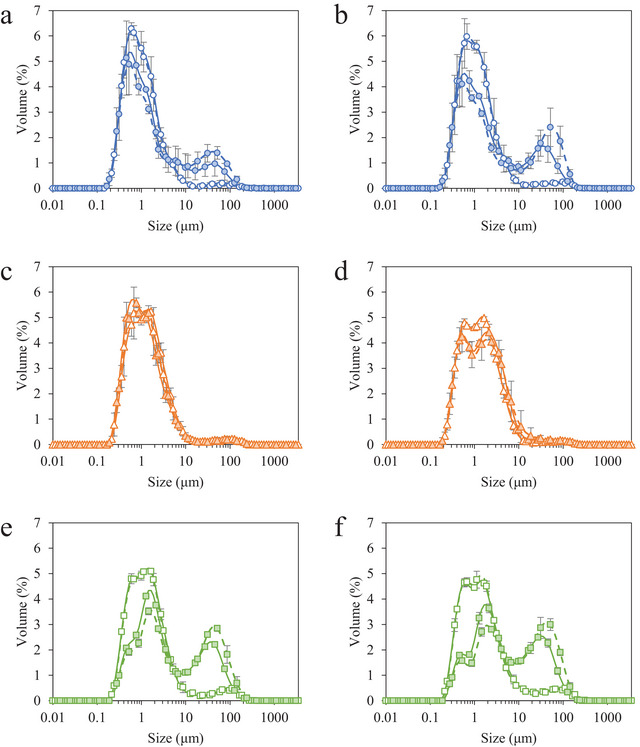
Fat globule size distributions for frozen desserts made with 6% milk protein concentrate (a, b), sodium caseinate (c, d), or whey protein isolate (e, f) after 0 weeks (a, c, e) and 6 weeks (b, d, f) of temperature‐cycled storage. Mono‐ and diglyceride addition of 0.0% (open symbols) or 0.15% (filled symbols) and overrun of 100% (solid lines) or 150% (dashed lines) are shown. Error bars represent the standard deviation of mean values measured in duplicate.

For frozen desserts made with MPC and WPI, the addition of 0.15% MDGs significantly increased the degree of fat destabilization, depicted as a peak in the range of 30–100 µm in the particle size distributions (Figure 4). Very little fat destabilization was observed in frozen desserts made with NaCN, and there were no significant effects of MDG addition, overrun, or storage time on the degree of fat destabilization (Table 3). Increasing overrun from 100% to 150% only showed a significant increase in fat destabilization for frozen desserts made with WPI and 0.15% MDGs. The apparent increase in fat destabilization with time was not significant for frozen desserts made with MPC, NaCN, or WPI.

**TABLE 3:** Fat destabilization of frozen desserts made with 6% milk protein concentrate (MPC), sodium caseinate (NaCN), or whey protein isolate (WPI) with varying levels of mono‐ and diglycerides (MDGs) and overrun (OR) measured during temperature‐cycled storage.

Fat destabilization (%)						
Protein source	MDGs (%)	OR (%)	Storage time (weeks)			
			0	2	4	6
MPC	0.0	100	4.73 ± 1.18^a,A,x^	5.04 ± 1.37^a,A,x^	4.23 ± 1.31^ab,A,x^	4.21 ± 1.86^a,A,x^
		150	4.26 ± 0.03^a,A,x^	6.00 ± 3.17^ab,A,x^	5.10 ± 1.10^a,A,x^	3.44 ± 1.35^a,A,x^
	0.15	100	19.4 ± 5.08^a,B,x^	22.2 ± 5.4^a,B,x^	20.4 ± 1.16^a,B,x^	26.9 ± 9.75^a,B,x^
		150	26.7 ± 3.49^a,B,y^	25.0 ± 4.3^a,B,x^	26.4 ± 3.56^a,B,y^	36.7 ± 11.9^a,B,x^
NaCN	0.0	100	3.53 ± 0.51^a,A,x^	3.00 ± 0.37^a,A,x^	3.32 ± 0.70^a,A,x^	3.27 ± 0.01^a,A,x^
		150	3.82 ± 0.70^a,A,x^	3.16 ± 0.10^a,A,x^	2.97 ± 0.77^a,A,x^	3.34 ± 0.45^a,A,x^
	0.15	100	3.87 ± 0.31^b,A,x^	4.29 ± 0.61^b,A,x^	3.99 ± 2.13^b,A,x^	4.13 ± 0.29^b,A,x^
		150	3.71 ± 1.99^b,A,x^	3.84 ± 1.31^b,A,x^	4.24 ± 1.44^b,A,x^	4.88 ± 0.43^b,A,x^
WPI	0.0	100	7.22 ± 0.16^a,A,x^	9.03 ± 0.63^a,A,x^	9.32 ± 0.57^b,A,x^	8.77 ± 0.58^a,A,x^
		150	8.66 ± 0.40^a,A,x^	10.9 ± 1.50^b,A,x^	13.7 ± 4.66^b,A,x^	9.49 ± 1.96^a,A,x^
	0.15	100	34.8 ± 1.09^c,B,x^	37.8 ± 5.71^c,B,x^	42.9 ± 2.64^c,B,x^	38.2 ± 5.42^a,B,x^
		150	45.2 ± 1.19^c,B,y^	48.8 ± 1.24^c,B,x^	47.3 ± 0.14^c,B,x^	51.2 ± 3.88^c,B,y^

*Note*: Mean ± SD are shown. Superscript letters a, b, and c denote significant differences with protein source; superscript letters A and B denote significant differences by MDG addition, and superscript letters x and y denote significant differences by overrun. Values in the same column sharing a letter are not significantly different at *α* = 0.05.

The development and stability of ice, air, and fat phases were strongly influenced by the formulation of the frozen desserts and functionality of protein and MDGs at interfaces and in the bulk serum phase. Interestingly, the development of the mix emulsion structure in the presence or absence of MDGs had a strong influence on the development of the air and ice phases, and the interaction between caseins, casein micelles, and serum proteins with polysaccharide stabilizers appeared to have the most significant influence on the stability of ice and air phases during storage.

The addition of MDGs significantly reduced the rate of ice crystal growth in frozen desserts made with MPC, WPI, and NaCN. The effects of MDGs were likely due to their effects on the surrounding fat, air, and serum phases. In the case of frozen desserts made with WPI and MPC, the addition of 0.15% MDGs increased the degree of fat destabilization due to competitive displacement of protein at the fat globule interface, thereby increasing the serum protein concentration. This combination of structural factors, including increased fat destabilization, increased serum protein concentration, and a small mean air cell size, reduced the mobility of water near the crystal surface and thus reduced the rate of ice crystal growth during storage.

In a mixed protein system such as MPC, specific protein–polysaccharide interactions, such as the complexation of serum proteins with galactomannans, electrostatic interactions of caseins with carrageenan, and incompatibility of specific globular or random coil proteins in solution, accelerated recrystallization in the high‐protein frozen desserts regardless of MDG addition or overrun. Previous studies have shown that freezing affects the viscoelasticity of aqueous serum proteins (Bhargava and Jelen 1995; Meza et al. 2010), which may have improved the synergy with locust bean gum and the strength and resilience of the protein gels in preventing recrystallization in frozen desserts made with WPI (Rocha et al. 2009; Silva et al. 2016).

Though there was no significant effect of MDGs on the fat destabilization in frozen desserts made with NaCN, the high viscosity of the protein‐concentrated serum phase likely reduced the rate of ice crystal growth during storage. Compared to native milk proteins, disordered caseins have been shown to increase the apparent viscosity of ice cream mixes (Huppertz et al. 2011), especially at low temperatures (Pitkowski et al. 2008). Additionally, the flexibility and hydrophobicity of β‐ and α_s1_‐casein promoted phase separation in the serum phase, while the flexibility of caseins enabled the bicontinuous matrix to withstand changes in osmotic pressure caused by temperature cycling.

In most frozen desserts, there was little effect of overrun on ice recrystallization. Thinner serum lamellae in frozen desserts made with 150% overrun prevented ice crystal growth during hardening, but the coarsening of the air phase and mobility of water in a protein‐depleted serum phase promoted recrystallization and resulted in a higher mean ice crystal size after 6 weeks compared to frozen desserts made with 0.15% MDGs.

Air cells stabilized by MPC have a viscoelastic character (VanWees 2024), but the mixture of proteins at air cell interfaces stabilized by MPC reduced the stability of the film (Coke et al. 1990; Mackie et al. 2001; Wilde et al. 2004), increasing the susceptibility to disproportionation and coalescence of air cells during storage. The destabilized networks in frozen desserts made with 0.15% MDGs, combined with the protein–polysaccharide phase separation in the serum phase, reduced drainage of serum and maintained a fine distribution of air cells in frozen desserts made with MPC and WPI, as observed previously (Chang and Hartel 2002a; Guo et al. 2018).

**FIGURE 5:**
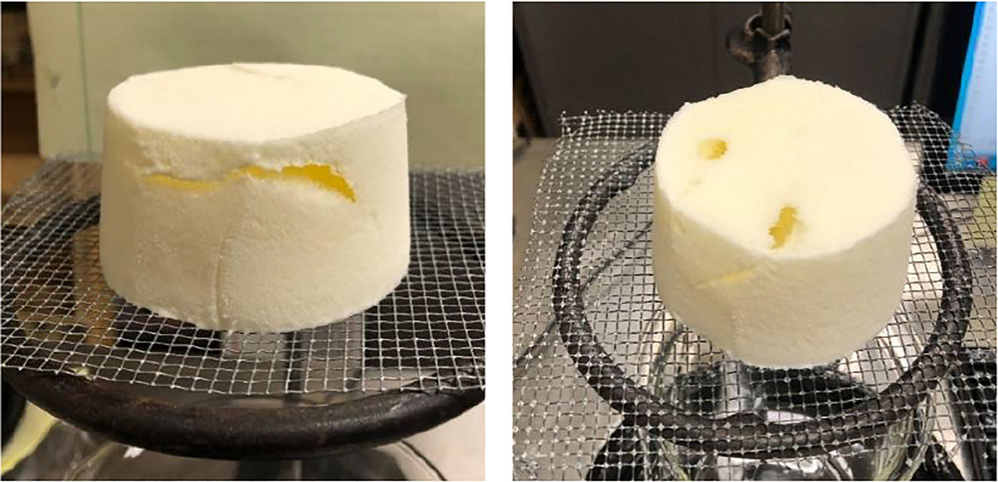
Channel formation observed in frozen desserts made with 6% sodium caseinate, 0.15% mono‐ and diglycerides, and 150% overrun after 2 weeks of temperature‐cycled storage.

The higher initial mean size and limited growth of air cells in frozen desserts made with NaCN may be explained by the viscoelasticity of caseins at the air interface. Flexible caseins adsorbed to the air interface and prevented the breakdown of air cells during the freezing process, even when 0.15% MDGs were added. The flexibility of the adsorbed caseins resisted severe volume changes during temperature‐cycled storage, especially in frozen desserts made with 0.0% MDGs, as the interfaces were primarily stabilized by caseins. The addition of 0.15% MDGs also promoted adsorption of fat globules to the interface (Buchheim and Dejmek 1990; Pelan et al. 1997), resulting in a mixed system with many incompatible mechanisms of stabilization (Brooker et al. 1986; Goff et al. 1999a; Zhang and Goff 2004, 2005). In particular, the co‐adsorption of caseins and MDGs resulted in structural collapse under dilatational stress (Barfod et al. 1991; Maldonado‐Valderrama and Rodríguez Patino 2010) and facilitated the diffusion of gas through the matrix, resulting in channel formation in frozen desserts made with 150% overrun after 2 weeks of storage (Figure 5). Though the increase in mean air cell size was relatively low, the macroscopic channels were evidence of severe air phase destabilization during temperature‐cycled storage and were not observed in any samples after freezing and hardening.

The structural integrity and high degree of fat destabilization in frozen desserts made with WPI and 0.15% MDGs resulted in a smaller mean air cell size and provided stability to the matrix to resist drainage, disproportionation, and coalescence. Conformational changes to the globular serum proteins upon adsorption (Dickinson 2003; Walstra et al. 2006) and the rupture of the film altered the structure and functionality of displaced serum proteins in the serum phase (Mackie et al. 2001; Zhang and Goff 2004). The increased viscosity of denatured serum protein dispersions likely improved the stability of air cells in the matrix during storage (Danesh et al. 2017; Lim et al. 2008; Rossa et al. 2012).

Fat destabilization occurs during dynamic freezing; the low temperatures and lack of shear during temperature‐cycled storage were not expected to affect the degree of fat destabilization in the frozen desserts studied. Interestingly, there was a slight increase in fat destabilization with storage, which may have been caused by the bridging, flocculation, or partial coalescence of existing structures caused by air, ice, and serum phase destabilization.

The addition of 0.15% MDGs significantly increased the fat destabilization in frozen desserts made with MPC and WPI due to the competitive displacement of proteins from the fat globule interface, as discussed previously (VanWees et al., forthcoming). The lower interfacial tension may also explain the apparent increase in fat destabilization during storage due to possible bridging or flocculation. The adsorption of flexible casein proteins resulted in strong, cohesive networks at the fat globule interface. Mono‐ and diglycerides were able to adsorb within these networks without displacing or disrupting the viscoelastic network that provided resistance to fat destabilization during freezing in frozen desserts made with NaCN. Emulsions stabilized by globular serum proteins were more susceptible to shear‐induced partial coalescence (Daw and Hartel 2015; Goff 1997; Goff et al. 1989; Segall and Goff 1999), especially when MDGs displaced serum proteins from the fat globule interface, as observed in frozen desserts made with WPI. Unlike frozen desserts made with MPC, the addition of MDGs did not significantly affect the degree of fat destabilization during storage, likely due to the composition of the interfacial and serum phases, which maintained the integrity of the matrix and facilitated its resistance to changes to the ice, air, and fat phases.

These findings suggest that the synergy between specific proteins and polysaccharide stabilizers effectively structures the serum phase and prevents recrystallization and air phase coarsening. The addition of 0.15% MDGs facilitated the destabilization of fat and an increase in serum protein concentration, establishing a scaffolding of destabilized fat to disrupt the flow of gas in the matrix and further reducing the mobility of water within pores of a polysaccharide gel network.

### 3.7 Melting Properties

**FIGURE 6:**
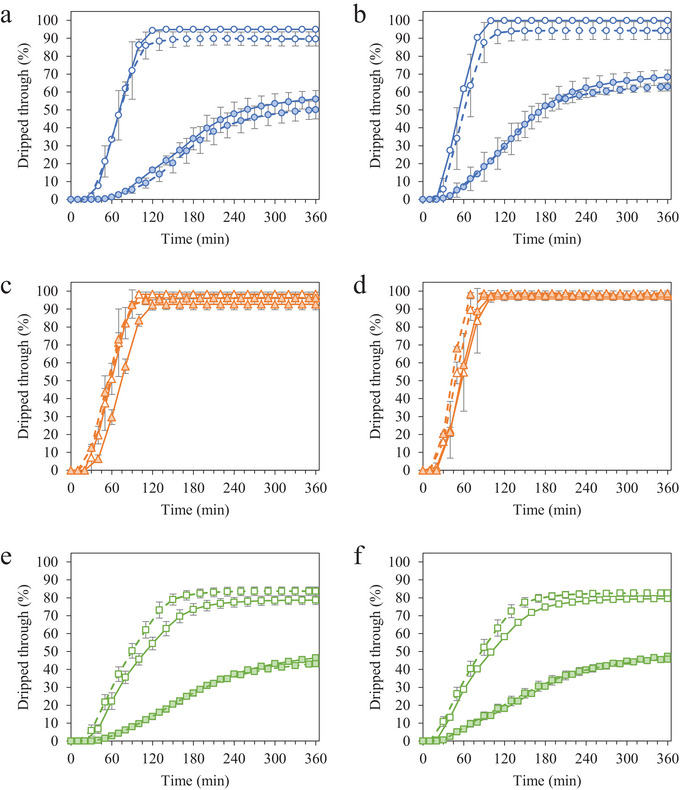
Melting behavior for frozen desserts made with 6% milk protein concentrate (a, b), sodium caseinate (c, d), or whey protein isolate (e, f) after 0 weeks (a, c, e) and 6 weeks (b, d, f) of temperature‐cycled storage. Mono‐ and diglyceride addition of 0.0% (open symbols) or 0.15% (filled symbols) and overrun of 100% (solid lines) or 150% (dashed lines) are shown. Error bars represent the standard deviation of mean values measured in duplicate.

Characteristic melting behavior was observed for frozen desserts made with MPC, NaCN, and WPI throughout storage (Figure 6), and select characteristics are explained in Table 4. The induction time, drip‐through rate, and shape retention of frozen desserts made with MPC and WPI were affected by MDG addition and overrun prior to storage, whereas all frozen desserts showed similar melting behavior. Overall, the collapse of the foam was accelerated as storage time increased, demonstrated by a decrease in induction time, an increase in drip‐through rate, and a decrease in shape retention for most frozen desserts studied.

**TABLE 4:** Melting properties of frozen desserts made with 6% milk protein concentrate (MPC), sodium caseinate (NaCN), or whey protein isolate (WPI) with varying levels of mono‐ and diglycerides (MDGs) and overrun (OR) measured during temperature‐cycled storage.

Induction time (min)						
Protein source	MDGs (%)	OR (%)	Storage time (weeks)			
			0	2	4	6
MPC	0.0	100	27.9 ± 1.92^a,A,x^	23.3 ± 0.97^a,A,x^	20.8 ± 0.89^a,A,x^	20.3 ± 1.35^a,A,x^
		150	26.0 ± 3.27^a,A,x^	17.7 ± 3.61^a,A,x^	18.0 ± 1.27^a,A,x^	23.5 ± 3.34^a,A,x^
	0.15	100	42.8 ± 0.34^a,B,x^	35.9 ± 0.656^a,B,x^	25.2 ± 0.20^a,A,x^	26.5 ± 0.59^a,A,x^
		150	46.9 ± 5.15^a,B,x^	29.1 ± 1.27^ab,B,x^	26.1 ± 0.93^a,A,x^	28.4 ± 6.63^a,A,x^
NaCN	0.0	100	22.9 ± 1.19^a,A,x^	22.9 ± 5.11^a,A,x^	21.0 ± 5.20^a,A,x^	19.9 ± 8.83^a,A,x^
		150	21.5 ± 5.18^a,A,x^	21.6 ± 0.95^a,A,x^	21.0 ± 2.84^a,A,x^	17.3 ± 2.22^a,A,x^
	0.15	100	30.5 ± 0.81^b,B,x^	29.8 ± 8.09^a,A,x^	27.2 ± 0.96^a,A,x^	23.8 ± 0.45^a,A,x^
		150	18.1 ± 1.98^b,B,y^	20.9 ± 2.34^a,A,y^	21.9 ± 5.78^a,A,x^	14.8 ± 0.19^b,A,y^
WPI	0.0	100	24.6 ± 0.92^a,A,x^	25.9 ± 1.27^a,A,x^	26.1 ± 2.58^a,A,x^	17.7 ± 1.04^a,A,x^
		150	21.1 ± 2.11^a,A,x^	18.4 ± 2.03^a,A,x^	21.9 ± 1.64^a,A,x^	15.6 ± 1.31^a,A,x^
	0.15	100	36.8 ± 3.79^ab,B,x^	37.5 ± 6.31^a,B,x^	41.7 ± 7.44^b,B,x^	26.0 ± 0.94^a,B,x^
		150	36.3 ± 0.87^c,B,x^	37.1 ± 3.18^b,B,x^	37.2 ± 8.19^b,B,x^	25.9 ± 2.28^a,B,x^

Drip‐through rate (% min^−1^)						
Protein source	MDGs (%)	OR (%)	Storage time (weeks)			
			0	2	4	6
MPC	0.0	100	1.39 ± 0.09^a,A,x^	1.47 ± 0.00^a,A,x^	1.66 ± 0.04^a,A,x^	1.67 ± 0.02^a,A,x^
		150	1.32 ± 0.22^a,A,x^	1.79 ± 0.07^a,A,y^	1.83 ± 0.04^a,A,x^	1.57 ± 0.12^a,A,x^
	0.15	100	0.311 ± 0.08^a,B,x^	0.385 ± 0.13^a,B,x^	0.496 ± 0.00^a,B,x^	0.394 ± 0.08^a,B,x^
		150	0.336 ± 0.07^a,B,x^	0.486 ± 0.06^a,B,x^	0.517 ± 0.01^a,B,x^	0.406 ± 0.00^a,B,x^
NaCN	0.0	100	1.61 ± 0.14^a,A,x^	1.60 ± 0.19^a,A,x^	1.59 ± 0.21^a,A,x^	1.67 ± 0.18^a,A,x^
		150	1.74 ± 0.23^b,A,x^	1.59 ± 0.04^a,A,x^	1.76 ± 0.24^a,A,x^	1.97 ± 0.18^b,A,y^
	0.15	100	1.39 ± 0.05^b,A,x^	1.62 ± 0.19^b,A,x^	1.79 ± 0.02^b,A,x^	1.79 ± 0.04^b,A,x^
		150	1.54 ± 0.05^b,A,x^	2.12 ± 0.31^b,B,y^	2.08 ± 0.15^b,B,y^	2.38 ± 0.05^b,B,y^
WPI	0.0	100	0.769 ± 0.05^b,A,x^	0.615 ± 0.07^b,A,x^	0.707 ± 0.04^b,A,x^	0.780 ± 0.03^b,A,x^
		150	0.792 ± 0.05^c,A,x^	0.695 ± 0.10^b,A,x^	0.691 ± 0.03^b,A,x^	0.760 ± 0.05^c,A,x^
	0.15	100	0.223 ± 0.00^a,B,x^	0.176 ± 0.03^a,B,x^	0.183 ± 0.01^c,B,x^	0.207 ± 0.00^a,B,x^
		150	0.218 ± 0.00^a,B,x^	0.187 ± 0.04^a,B,x^	0.188 ± 0.03^c,B,x^	0.211 ± 0.01^a,B,x^

Dripped‐through (%)						
Protein source	MDGs (%)	OR (%)	Storage time (weeks)			
			0	2	4	6
MPC	0.0	100	95.0 ± 0.61^a,A,x^	94.9 ± 0.84^a,A,x^	99.8 ± 0.16^a,A,x^	99.9 ± 0.05^a,A,x^
		150	89.6 ± 3.92^ab,A,x^	97.9 ± 0.45^a,A,x^	99.5 ± 0.07^a,A,x^	94.3 ± 4.92^a,A,y^
	0.15	100	56.0 ± 4.78^a,B,x^	60.9 ± 6.01^a,B,x^	70.7 ± 0.84^a,B,x^	68.4 ± 3.91^a,B,x^
		150	50.1 ± 5.03^a,B,x^	65.2 ± 2.06^a,B,x^	67.3 ± 0.25^a,B,x^	62.9 ± 2.25^a,B,y^
NaCN	0.0	100	98.4 ± 0.99^a,A,x^	97.9 ± 2.94^a,A,x^	96.2 ± 2.96^a,A,x^	97.5 ± 2.69^a,A,x^
		150	96.1 ± 3.39^a,A,x^	95.7 ± 0.23^a,A,x^	95.3 ± 1.75^a,A,x^	97.8 ± 0.45^a,A,x^
	0.15	100	92.7 ± 3.06^b,A,x^	93.6 ± 5.29^b,A,x^	95.1 ± 1.78^b,A,x^	97.6 ± 0.42^b,A,x^
		150	96.3 ± 1.62^b,A,x^	95.6 ± 0.81^b,A,x^	96.7 ± 3.07^b,A,x^	98.8 ± 0.39^b,A,x^
WPI	0.0	100	78.7 ± 2.47^b,A,x^	72.1 ± 3.50^b,A,x^	73.2 ± 0.37^b,A,x^	79.5 ± 0.13^b,A,x^
		150	83.7 ± 2.41^b,A,x^	79.6 ± 5.34^b,A,x^	78.7 ± 1.14^b,A,x^	82.6 ± 1.81^b,A,x^
	0.15	100	46.4 ± 0.30^c,B,x^	38.8 ± 6.67^c,B,x^	38.8 ± 3.01^c,B,x^	47.2 ± 0.47^c,B,x^
		150	43.2 ± 1.55^a,B,x^	34.3 ± 2.10^c,B,x^	38.1 ± 6.62^c,B,x^	45.6 ± 1.27^c,B,x^

*Note*: Mean ± SD are shown. Superscript letters a, b, and c denote significant differences with protein source; superscript letters A and B denote significant differences by MDG addition; and superscript letters x and y denote significant differences by overrun. Values in the same column sharing a letter are not significantly different at *α* = 0.05.

Frozen desserts made with NaCN had the lowest induction times overall, and the induction time for frozen desserts made with MPC and WPI increased when 0.15% MDGs were added due to the presence of destabilized fat and gelation of polysaccharides and/or proteins in the serum phase. Ice crystal size had no significant effects on initial induction time, but all frozen desserts took less time to melt following temperature‐cycled storage.

Induction time decreased for all frozen desserts during storage, with the decrease being significant for those made with MPC and WPI. While the phase separation of proteins and polysaccharides inhibited the molecular mobility of water, gelation of locust bean gum decreased the viscosity of the surrounding aqueous phase due to syneresis (Bourriot et al. 1999; Regand and Goff 2002). Melting of ice further diluted this liquid phase, and the surface of the product began dripping more quickly, thus increasing the induction time recorded for the frozen desserts. The continued dilution of the aqueous phase during temperature‐cycled storage accelerated the induction time, especially in frozen desserts with a higher concentration of aqueous protein, such as those containing 0.15% MDGs. Frozen desserts made with NaCN had the fewest changes in ice, air, and fat structures during storage, and though the induction time decreased significantly with storage, the rate of change was not affected by MDG addition or overrun.

The drip‐through rate was strongly affected by the frozen dessert formulation, with some formulations melting more quickly following temperature‐cycled storage (Table 4). Drip‐through rate was fastest for frozen desserts made with NaCN and slowest for those made with WPI. For frozen desserts made with MPC and WPI, the addition of 0.15% MDGs resulted in destabilized fat networks that ultimately reduced the flow of the serum phase and stabilized the foam (Baer et al. 1997; Bolliger et al. 2000; Cropper et al. 2013; Koxholt et al. 2001).

For frozen desserts made with MPC, the drip‐through rate increased with storage time, likely due to the coarsening of the ice and air phases during temperature‐cycled storage, especially in frozen desserts made with 0.0% MDGs. Frozen desserts made with WPI had the highest degree of fat destabilization and the fewest changes to ice, air, and fat phases during storage, so the limited change in drip‐through rate with storage was not unexpected. The drip‐through rate increased significantly with storage in frozen desserts made with NaCN. The formation of visible air channels severely disrupted the structure of frozen desserts made with NaCN, 0.15% MDGs, and 150% overrun, resulting in a much faster collapse of the foam and an increased drip‐through rate compared to samples with less observable air phase destabilization.

Frozen desserts made with NaCN, as well as those made with MPC and 0.0% MDGs, had very little shape retention following melting, whereas frozen desserts made with WPI had some foam remaining after 6 h. When 0.15% MDGs were added to frozen desserts made with MPC and WPI, frozen desserts had significantly more shape retention than those with 0.0% MDGs, while overrun had a limited effect. A greater fraction of frozen dessert dripped through the screen following storage for frozen desserts made with MPC, especially without added MDGs, but there were no significant effects of storage time for frozen desserts made with NaCN or WPI.

The effects of temperature‐cycled storage on ice, air, and serum phases in frozen desserts made with MPC were evident in the melting behavior. Unexpectedly, the drip‐through rate of frozen desserts made with MPC and 0.15% MDGs was higher following storage despite the structuring role of destabilized fat networks. This was not observed in frozen desserts made with WPI, which had some degree of fat destabilization, suggesting that the increase in drip‐through rate was related to the functionality of casein and micellar casein. Wu (2023) noted significant phase separation in model systems containing nonfat dry milk and polysaccharides, which influenced the melting behavior of the frozen model systems. The decrease in induction time and shape retention and the increase in drip‐through rate were likely related to the phase separation of the serum phase, and this collective degradation of ice, air, and serum structures during storage resulted in weaker structural retention during melting.

### 3.8 Shrinkage

All frozen desserts had some loss of product volume due to the destabilization of the initial ice, air, fat, and serum phases, yet only frozen desserts made with NaCN, 0.15% MDGs, and 150% overrun experienced significant shrinkage during the 6 weeks of temperature‐cycled storage (Table 5). These frozen desserts had significant shrinkage after 2 weeks and continued to shrink with further storage. The addition of MDGs decreased the rate of shrinkage in frozen desserts made with MPC but not in those made with WPI. Overrun had no significant effects for those made with MPC or WPI.

**TABLE 5:** Shrinkage (percentage of volume lost) of frozen desserts made with 6% milk protein concentrate (MPC), sodium caseinate (NaCN), or whey protein isolate (WPI) with varying levels of mono‐ and diglycerides (MDGs) and overrun (OR) measured during temperature‐cycled storage.

Shrinkage (%)						
Protein source	MDGs (%)	OR (%)	Storage time (weeks)			
			0	2	4	6
MPC	0.0	100	NS	2.82 ± 2.10^a,A,x^	1.83 ± 3.50^a,A,x^	0.84 ± 3.50^a,A,x^
		150	NS	3.56 ± 1.05^a,A,x^	1.58 ± 2.45^a,A,x^	0.59 ± 1.05^a,A,x^
	0.15	100	NS	3.06 ± 3.85^a,A,x^	2.82 ± 2.10^a,A,x^	3.61 ± 0.98^a,A,x^
		150	NS	2.57 ± 0.35^a,A,x^	2.07 ± 1.05^a,A,x^	5.54 ± 0.35^a,B,x^
NaCN	0.0	100	NS	2.92 ± 0.56^a,A,x^	0.84 ± 3.50^a,A,x^	2.82 ± 2.10^a,A,x^
		150	NS	5.09 ± 0.28^a,A,x^	1.09 ± 2.45^a,A,x^	3.66 ± 1.19^a,A,x^
	0.15	100	NS	1.21 ± 0.17^a,A,x^	0.34 ± 1.40^a,A,x^	1.18 ± 1.05^a,A,x^
		150	NS	14.6 ± 3.64^b,B,y^	14.7 ± 2.10^b,B,y^	32.0 ± 1.40^b,B,y^
WPI	0.0	100	NS	0.59 ± 3.15^a,A,x^	2.32 ± 1.40^a,A,x^	1.53 ± 3.08^a,A,x^
		150	NS	1.18 ± 0.21^a,A,x^	6.28 ± 1.40^a,A,x^	4.30 ± 1.40^a,A,x^
	0.15	100	NS	0.59 ± 1.05^a,A,x^	2.32 ± 1.40^a,A,x^	1.53 ± 0.280^a,A,x^
		150	NS	3.56 ± 1.05^a,A,x^	5.09 ± 0.28^a,A,x^	3.31 ± 2.80^a,A,x^

*Note*: Mean ± SD are shown. Superscript letters A and B denote significant differences by MDG addition; superscript letters x and y denote significant differences by overrun; and superscript letters a, b, c, and d denote significant differences with storage time. Values sharing a letter are not significantly different at *α* = 0.05.

Abbreviation: NS, no shrinkage.

**FIGURE 7:**
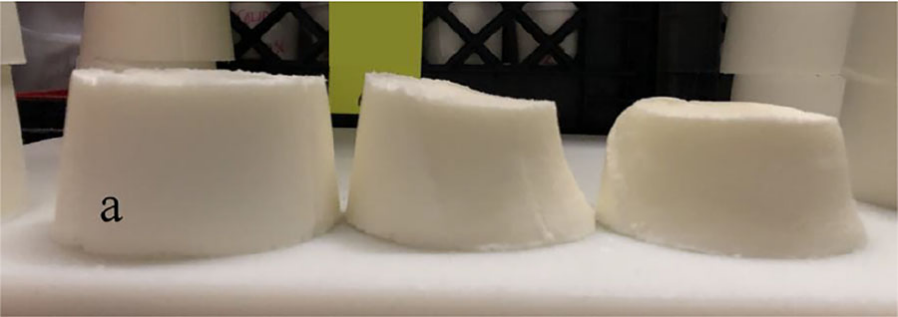
Shrinkage observed in frozen desserts made with 6% sodium caseinate, 0.15% mono‐ and diglycerides, and 150% overrun following temperature‐cycled storage. Sample “a” depicts a fresh (0 weeks of storage) sample.

Visible shrinkage occurred in three dimensions (Figure 7); the product collapsed under the weight of gravity and pulled away from the sides and top of the container. Frozen desserts were hardened and stored upside down during storage, so the concave appearance at the top of the container shown was not a result of drainage or gravitational collapse but rather coalescence and disproportionation of air cells and other factors that caused the product to recede from the top of the container.

Despite the significant effects of protein source, MDG addition, overrun, and temperature‐cycled storage on the structural integrity of the frozen desserts, shrinkage was only significant in frozen desserts made with NaCN, 0.15% MDGs, and 150% overrun. In this formulation, the flexible, fluid‐like structure in the interface enabled the diffusion of gas and channel formation, which is known to precede product collapse and shrinkage (Goff et al. 1995; Turan et al. 1999). In addition to the low viscoelasticity and limited intermolecular interfacial interactions between caseins, adsorption of MDGs to the air interface further decreased the stability of the air phase. The mixed NaCN–MDG interface was less stable than a pure protein‐ or MDG‐stabilized system, and the film was prone to deformation and collapse (Coke et al. 1990; Maldonado‐Valderrama et al. 2007). The mechanisms of channel formation by air cell coalescence or disproportionation, caused by weakened internal structures and/or interfacial instability, may also directly influence the shrinkage of frozen desserts, even if the internal channels are not visible.

Though the barrier to displacement by small molecule surfactants is higher for planar monolayers of β‐lactoglobulin compared to β‐casein (Mackie et al. 2001), there was significantly less air phase coarsening in frozen desserts made with WPI or MPC. Furthermore, air cells in high‐overrun frozen desserts are susceptible to destabilization via gas diffusion in the thinner lamellae (Goff et al. 1999b; Jiang et al. 2019; Sommer 1946). Destabilization by coalescence, disproportionation, and drainage occurred during temperature‐cycled storage due to the lack of stability in the serum phase, low‐fat destabilization, and weak interfacial networks. Poorly viscoelastic caseins had a low degree of cross‐linking and were further destabilized by the addition of incompatible MDGs, which enabled the diffusion of gas through the serum and accelerated channel formation and shrinkage in frozen desserts made with NaCN, 0.15% MDGs, and 150% overrun.

### 3.9 Stabilization of Complex, Multiphase Foams During Melting and Storage

The aim of this work was to elucidate a possible mechanism of shrinkage by studying the effect of specific microstructural elements (e.g., ice crystals, air cells, and fat networks) on foam collapse at room temperature (melting behavior) and during storage (shrinkage) to further explain the stepwise formation and destabilization of the foam.

Multiple linear regression and similar statistical techniques have previously been used to correlate ice, air, and fat structures with melting behavior in frozen desserts (Amador et al. 2017; Freire et al. 2020; Muse and Hartel 2004; Warren and Hartel 2018; Wu et al. 2019). Fat destabilization is often the most significant predictor of melting behavior, as was observed in this study; however, the changes in ice and air phases during storage provided additional insight into foam collapse at room temperature.

**FIGURE 8:**
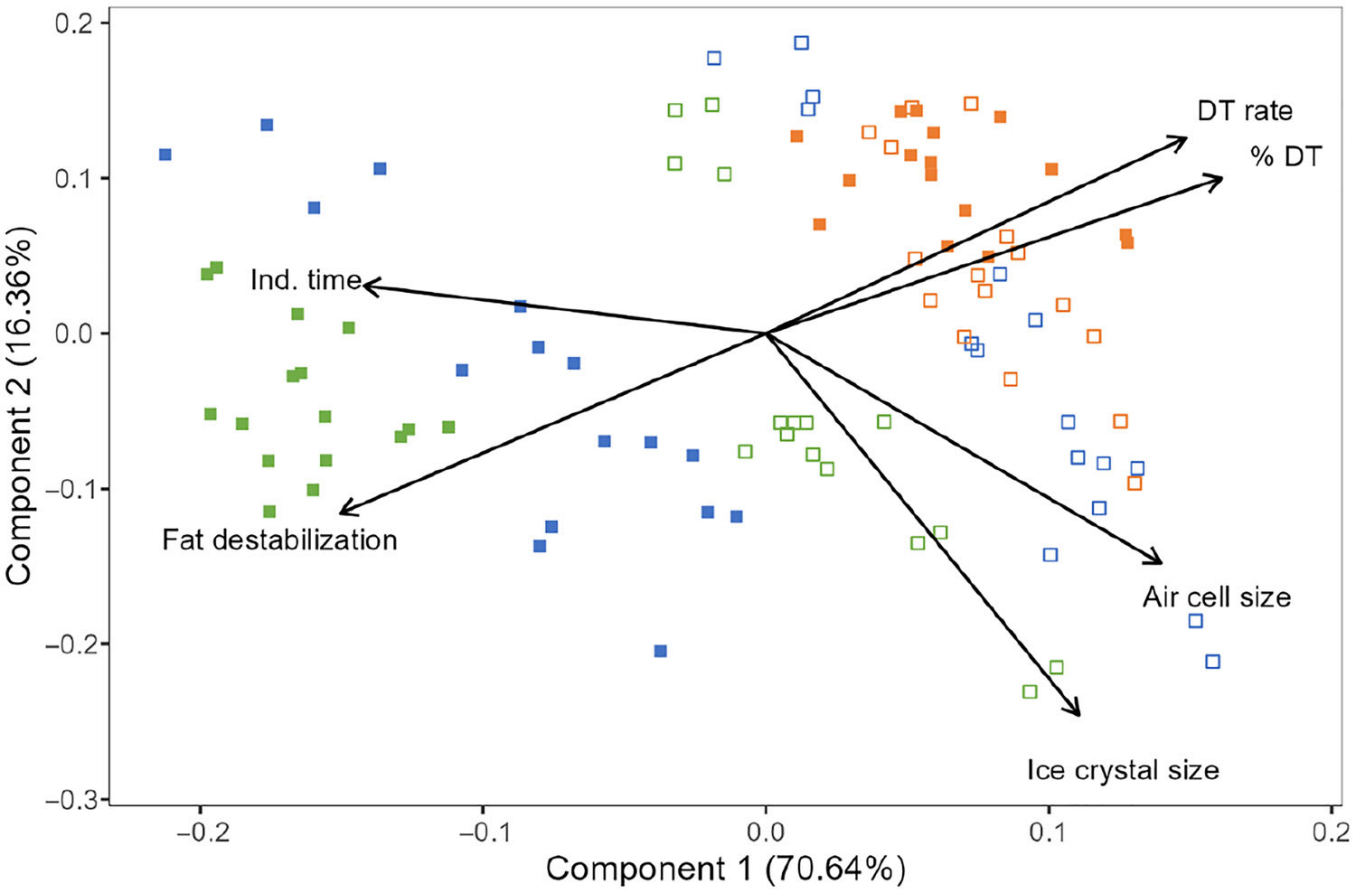
Principal component biplot for melting and microstructural properties of frozen desserts made with milk protein concentrate (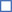 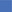), sodium caseinate (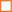 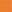), and whey protein isolate (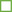 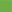). Mono‐ and diglyceride addition of 0.0% (open symbols) or 0.15% (filled symbols) is shown for all overrun conditions and storage times. Ind. time = induction time; DT rate = drip‐through rate; % DT = percentage dripped through after 360 min of melting.

Principal component analysis of the microstructural and melting parameters showed clear associations between formulation, structure, and melting properties (Figure 8). The extent of fat destabilization was negatively correlated with drip‐through rate and the percentage of frozen dessert that dripped through the screen, whereas mean ice crystal and air cell sizes were not strongly related to any melting properties. Changes to induction time, drip‐through rate, and shape retention during temperature‐cycled storage were likely associated with the overall coarsening of the ice and air phases, the initial formulation and structure, and changes in serum phase composition and structure (e.g., phase separation, gelation, lamellar thickness) rather than individual microstructural elements.

**FIGURE 9:**
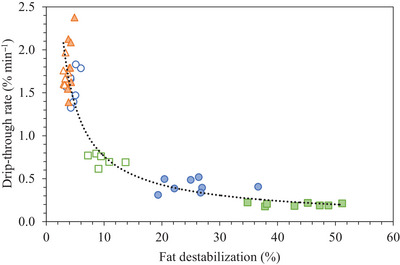
Drip‐through rate plotted as a function of fat destabilization for frozen desserts made with 6% milk protein concentrate (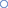,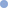), sodium caseinate (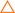,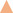), or whey protein isolate (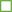,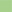) (*R* ^2^ = 0.847). Mono‐ and diglyceride addition of 0.0% (open symbols) or 0.15% (filled symbols) is shown. Error bars of duplicate measurements are omitted for clarity.

When all frozen desserts were considered, there was a significant increase in induction time with greater fat destabilization and lower mean ice crystal and air cell sizes. There was a strong correlation between fat destabilization and drip‐through rate, but mean ice crystal or air cell sizes had no effect. The relationship between drip‐through rate (DT rate) and fat destabilization (FD) is shown in Figure 9 and detailed in Equation (2):

(2) $$\mathrm{DT}\;\mathrm{rate}=5.12{\left(\mathrm{FD}\right)}^{-0.828}.$$

Like the drip‐through rate, the shape retention of the frozen desserts was strongly affected by fat destabilization in frozen desserts made with MPC and WPI, but not in those made with NaCN. As ice crystal size increased due to recrystallization, the weakened foam matrix was unable to resist collapse during melting, and shape retention decreased. The effects of structure were most prevalent in frozen desserts made with WPI, where fat destabilization, ice crystal size, and air cell size showed significant correlations with shape retention in frozen desserts made with WPI. The shape retention of frozen desserts with MPC was weakly affected by the size of ice crystals and air cells, but there were no effects in frozen desserts made with NaCN.

Six weeks of temperature‐cycled storage at these conditions was not sufficient to induce significant shrinkage in most of the frozen desserts in this study. Shrinkage was not strongly associated with ice crystal size or fat destabilization, but increasing air cell size did correlate with an increase in shrinkage in the 6 weeks of storage. The poor linear model fit for each individual protein system (*R* ^2^ = 0.25–0.29) demonstrated the complexity of frozen desserts and the challenges in predicting shrinkage based on individual structural measurements. While 6 weeks was not long enough to induce significant shrinkage in most of the frozen desserts, all samples demonstrated apparent shrinkage after 3 months of storage under similar conditions (data not shown).

In the case of both melting and shrinkage, the shape of the product is retained by the properties of the foam matrix; however, no correlation was observed between shape retention during melting and shrinkage (*r* = 0.152).

### 3.10 Possible Mechanisms of Shrinkage

The destabilization and collapse of the frozen foam primarily depends on the composition and rheological properties of the air cell interface, the composition and rheological properties of the foam matrix phase, and the ability of the matrix to withstand temperature and/or pressure fluctuations (VanWees et al. 2022). In this study, the shrinkage of high‐protein, high‐overrun frozen desserts was reduced in products in which structural integrity was maintained during abusive storage to prevent air phase destabilization. Previous research has also shown that air cell interfaces stabilized by WPI had higher dilatational viscoelastic moduli but were prone to rupture at high stress, whereas interfaces stabilized by NaCN were more fluid‐like and resilient, demonstrating the complexity of air phase stabilization in milk protein systems (VanWees 2024).

Shrinkage is thermodynamically favorable and will proceed unless there are sufficient barriers to disproportionation, coalescence, and drainage of the dispersed air phase. This experiment showed that optimizing the solid‐like properties of the frozen dessert through formulation and processing was the most effective means of preventing shrinkage during storage at fluctuating temperatures. A scaffold of destabilized fat networks that bridged the lamellar distance between air cells provided resistance to drainage, while the likely phase separation of proteins and polysaccharides reduced diffusion of gases through pores in the matrix. Ensuring a distribution of small ice crystals and air cells during freezing was conducive to extending the shelf life of the product and reducing the extent of ice and air phase destabilization during storage, thereby reducing the susceptibility and rate of shrinkage. Of the protein sources studied, sources with micellar caseins and/or serum proteins had the most synergy with added MDGs to optimize the fat, ice, air, and serum structures and prevent shrinkage in high‐protein frozen desserts.

## 4 Conclusion

The stepwise development of structure and the complexity of the multiphase systems are key to understanding mechanisms of destabilization and collapse of frozen desserts. Destabilized fat networks provided structure to the foam during melting by entrapping air and liquid in the melted foam and reducing the drip‐through rate. Fat destabilization was highest when MDGs were added due to the displacement of protein from the interface, though the displacement was dependent on the structure of the dairy protein used. There were a few changes to the fat phase during storage, but the effects of protein displacement and fat structures in the product were key to understanding the coarsening of ice and air phases during temperature‐cycled storage. In general, the concentrated serum phase prevented the mobility of water and gas, maintaining the solid‐like structure of the matrix and preventing ice recrystallization and air cell coarsening. The mechanisms of action in the phase‐separated serum phase were dependent on the type of protein, but frozen desserts made with MDGs generally had fewer changes to ice and air phases due to the concentrated serum phase and presence of fat networks.

The interplay between ice, air, fat, and serum phases provided critical insight into the mechanisms of shrinkage, which is best described as a collapse of the frozen foam. Air phase destabilization was greatest in products that had a weak interfacial structure, low fat destabilization, and low serum protein structuring, resulting in channeling of air cells and a collapse of the foam at room temperature and during storage. Predicting shrinkage during temperature‐cycled storage is challenging given the complexity of the product matrix, but shrinkage can be prevented by optimizing the structure of the air interface and frozen dessert matrix through careful manipulation of ingredients, processing, and storage.

## Nomenclature

MDG: mono‐ and diglyceride
MPC: milk protein concentrate
NaCN: sodium caseinate
WPI: whey protein isolate

## Author Contributions

**Samantha R. VanWees**: conceptualization, methodology, data curation, investigation, formal analysis, visualization, writing – review and editing, writing – original draft. **Scott A. Rankin**: funding acquisition, writing – review and editing, project administration, supervision, conceptualization, validation. **Richard W. Hartel**: conceptualization, validation, supervision, writing – review and editing, funding acquisition, project administration.

## Conflicts of Interest

The authors declare no conflicts of interest.

## Data Availability

Data are available upon reasonable request.
